# Smoking cessation counselling training in the pre-clerkship curriculum of Canadian medical schools: A national survey

**Published:** 2018-05-31

**Authors:** Matthew Loranger, Kayla Simms, Andrew Pipe

**Affiliations:** 1 Faculty of Medicine, University of Ottawa, Ontario, Canada; 2 Division of Prevention & Rehabilitation, University of Ottawa Heart Institute, Ontario, Canada

## Abstract

**Background:**

Cigarette use is Canada’s leading cause of preventable disease, disability, and death. The Medical Council of Canada requires that physicians be able to address tobacco-use, however smoking cessation counselling (SCC) training remains largely neglected in the pre-clerkship curricula of many Canadian medical schools.

**Methods:**

Between July and October of 2015, Canada’s 17 medical schools were invited to participate in an administrative survey: *The Canadian Medical School Assessment of Smoking Cessation Counselling in the Pre-Clerkship Curriculum*. Each was asked to comment on the presence of 28 tobacco-related topics in their curricula, including: time devoted to source material; year(s) of training during which medical students were exposed to related content; methods of teaching and examination; and, the attitudes, policies, and barriers relevant to the presence of smoking cessation counselling (SCC) training in the curriculum.

A second short survey: *Assessing Medical Students Attitudes toward Smoking Cessation Education* was distributed to 100 University of Ottawa medical students to assess comfort level and perceived confidence toward addressing smoking cessation with patients.

**Results:**

Eleven of 17 medical schools completed the administrative survey. The results demonstrated substantial deficits and inconsistencies in the delivery of SCC training in the pre-clerkship curricula of Canada’s medical schools. The short survey revealed perceived discomfort regarding smoking cessation discussion, consistent with the potential curriculum deficits suggested in the larger national survey.

**Conclusion:**

The results of both surveys suggest an unfortunate oversight given the devastating impact of tobacco-related diseases. Institutional commitment and enhanced inter-university collaboration could facilitate the development of a national undergraduate medical education program to enhance the delivery of SCC training within the pre-clerkship curricula of Canadian medical schools.

## Introduction

Cigarette use is Canada’s leading cause of preventable disease, disability and death. The World Health Organization (WHO) estimates that 1.3 billion smokers purchase 10 million cigarettes every minute around the globe, that every eight seconds someone dies from a tobacco-related disease, and that six million smokers will die annually as a result of tobacco addiction.^1,2^ Despite a national decline in smoking prevalence, Statistics Canada estimates that 18.1% of Canadians are currently smokers.^3^ Clinical interventions, involving counselling and pharmacotherapy, can effectively assist in initiating and sustaining smoking cessation.^4-7^ Canadian smokers will lose at least a decade of life-expectancy, but if cessation can be achieved by age 40, at least 90% of the predicted premature mortality can be prevented.^8^ Physicians and physicians-in-training, have particular responsibilities and opportunities to address nicotine addiction in clinical settings. The systematic delivery of appropriate, evidence-based, best-practice smoking cessation interventions by clinicians would be of substantial benefit to their patients and the community. Epidemiologic data and clinical realities suggest that approaches to the treatment of nicotine addiction are fundamental skills for Canada’s medical students.^9^ To understand current educational practice regarding training in smoking cessation counseling (SCC) and to better plan for relevant educational programming, such as Motivational Interviewing (MI), we completed a national survey of relevant curricular content in Canada’s medical schools.

## Methods

Between July and October of 2015, all 17 Canadian medical schools were invited to participate in an administrative survey entitled: Canadian Medical School Assessment of Smoking Cessation Counselling in the Pre-Clerkship Curriculum. The survey was developed by adapting and modifying similar questionnaires used previously in national settings.^10-12^ The survey assessed the presence in the undergraduate curriculum of 28 tobacco-related topics (Table 1). Medical schools were asked to respond indicating: the time devoted to SCC training; the year(s) in which such training took place; methods of instruction and examination; and, attitudes, barriers, and policies concerning the inclusion of SCC training in the curriculum. Undergraduate medical education faculty were identified from each of the 17 Canadian medical school websites and contacted directly via personalized email messages, which contained the survey. In the email they were prompted to forward the survey to the most appropriate faculty member regarding expertise in smoking cessation related curriculum at their institution. Subsequently, up to three follow-up reminder emails were sent at three-week intervals. Institutional responses are highlighted in Table 2. A separate short survey entitled “Assessing Medical Students Attitudes toward Smoking Cessation Education at the University of Ottawa” was distributed to 100 University of Ottawa medical students in the cohorts graduating between 2016-2018, who had completed a minimum of one year of medical school. Students were recruited using the Facebook class-sites for each of the three cohorts. The first 100 students to respond were included. The survey consisted of a questionnaire designed to assess medical students’ perception of smoking cessation education at the University of Ottawa using questions with a 5-point scale. Their responses are highlighted in Table 3. The survey instruments for each questionnaire were distributed using survey monkey (www.surveymonkey.com); they were developed, and the results analyzed, by the authors. Both surveys are available upon request.

**Table 1:** Number of medical schools reporting coverage of knowledge and skills related to smoking cessation and tobacco dependence

Epidemiology of tobacco addiction	10
Cardiovascular risk	9
Second-hand smoke	9
Available smoking cessation programs/community resources	9
Impact of public policies on smoking	9
COPD risk	9
Cancer risk	9
Smoking and other substances (i.e., alcohol, marijuana, amphetamines)	9
The role of smoking cessation services	8
Smoking and pregnancy	8
Nicotine replacement therapy	8
Harm reduction	8
Cost and clinical effectiveness of stop smoking interventions	8
Teratogenicity of smoking	7
Nicotine withdrawal symptoms	7
Pharmacology of nicotine addiction	7
Other pharmacological agents	6
Advocacy for tobacco control	6
Smoking and Indigenous health	6
Smoking and mental health	5
Contents of cigarette smoke	5
Benefits of cessation prior to surgery	5
Harmful effects of related products (i.e., hookah, e-cigarettes)	4
Practical delivery in artificial settings (i.e., role play)	4
Third-hand smoke	3
Practical delivery in clinical settings (i.e., shadowing practitioner)	3
Smoking and homelessness	2
Cigarette design	2

**Table 2:** Smoking Cessation Counselling Training (SCCT) content in the pre-clerkship curriculum: individual responses of Canadian medical schools

Medical School	SCCT covered in pre-clerkship	Number of hours devoted	Use of Didactic Lectures	Enhanced Methods of Learning	Use of Counselling Session (role-play, real or standardized patients)
McGill University	No*	0	No	No	No
Memorial University	Yes	1-3	Yes	No	No
Northern Ontario School of Medicine	Yes	1-3	No	CBL, small groups	No
University of Alberta	Yes	3-5	Yes	CBL, PBL,	No
University of British Columbia	Yes	>5	Yes	CBL, PBL,	Yes
University of Calgary	Yes	3-5	Yes	CBL, Small Group Sessions	No
University of Manitoba	Yes	3-5	Yes	Small Group Sessions	Yes
University of Montreal	Yes	>5	Yes	PBL, Small Group Session, SLM	Yes
University of Ottawa	Yes	1-3	Yes	No	No
University of Saskatchewan	Yes	1-3	Yes	PBL, Small Group Sessions	No
Queen’s University	Yes	1-3	Yes	TBL	No

* SCCT was not introduced until the clerkship curriculum (years 3-4).

**Table 3:** Percentage of medical students responding to assessment of attitudes toward Smoking Cessation Counselling (SCC) education at the University of Ottawa (N = 100)

Responders in 2018 graduating cohort*	37
Responders in 2017 graduating cohort	33
Responders in 2016 graduating cohort	30
Have attempted to counsel patients in smoking cessation	44
Believe SCC competency is essential to being a competent physician	67
Believe it is very important SCC training appear in the pre-clerkship curriculum	80
Feel capable and comfortable addressing smoking cessation with patients	11

* Students in 2018 graduating cohort had completed the 1^st^ year of medical school at the time of the survey

## Results

Of 17 institutions, 11 medical schools completed their survey. One school formally declined participation and five schools did not respond.

Multiple departments were reported to be involved in teaching within the 11 responding schools: Public Health, Respirology, Family Medicine and Internal Medicine were most commonly identified. Smoking and tobacco-related content was always delivered by faculty members; none of the institutions reported the use of external lecturers in addressing smoking and tobacco-related issues. SCC training appeared in the third year curriculum in six of the 11 schools and in the fourth year in three other schools. Knowledge of smoking cessation related content was formally examined, primarily by multiple choice and/or a written examination, in seven of the schools. At only three schools were counselling skills evaluated by a physician or nurse practitioner during pre-clerkship training. No institutions reported use of practice or mock Objective Structured Clinical Examinations (OSCEs) to assess SCC competency.

Instructional approaches varied. Nine schools reported incorporating large group session lecture(s), five of whom supplemented this form of instruction with Case or Problem Based Learning (CBL/PBL). Two schools used Team Based Learning (TBL) and one used Physician Skills Development (PSD) to address SCC training. Observation of counselling skills was reported in only three of the 11 schools; two of which used simulated role-play interaction. One institution reported the use of a Self Learning Module (SLM).

Only three schools identified academic barriers to facilitating SCC training: one cited crowding of the curriculum as an issue; two schools identified a general lack of enthusiasm among the faculty; and four institutions claimed funding was insufficient to support it.

### Results of the medical student survey

One hundred medical students at the University of Ottawa completed the survey entitled “Assessing Medical Students Attitudes toward Smoking Cessation Education at the University of Ottawa”. Of those surveyed, the majority were in the cohort graduating in 2018 and had completed one year of medical school; 44 claimed they had experience counselling patients in smoking cessation; 67 believed SCC competence was essential to being a competent physician; 80 believed it should appear in the pre-clerkship curriculum. Only a minority (11) felt they were capable of and comfortable addressing smoking cessation with patients.

## Discussion

Although the Medical Council of Canada specifies in its objectives that physicians be able to manage smoking cessation, SCC training does not appear to receive sufficient emphasis in the pre-clerkship curricula of many Canadian medical schools to adequately address this objective.^9^

Raupach and colleagues in 2015 identified substantial deficits in the SCC training of U.K. medical students – a reality that remained largely unchanged over a decade.^10,13^ Internationally, as in Canada, there appears to be a dearth of SCC education within medical school curricula.^11,14-16^

The voluntary survey of 100 medical students at the University of Ottawa (Table 3) revealed that only a small minority expressed comfort in addressing smoking cessation with patients. This self-acknowledged discomfort with addressing smoking cessation is concerning and merits a re-examination of curricula content and delivery of SCC training.

Strategies such as MI have been broadly emphasized in the practical and clinical skills training of medical students and have been shown to enhance confidence and knowledge regarding approaches to behavioural intervention.^17^ A combination of patient-centered counselling and evidence-based pharmacotherapies produces significantly increased abstinence rates when treating nicotine addiction – and might usefully form the basis of SCC training in the pre-clerkship years.^6,7^

Although the majority of Canadian institutions that participated in the curriculum survey claimed to promote knowledge of pharmacological interventions and employ one or more enhanced methods of learning including, but not limited to, CBL, PBL, TBL, PSD and SLMs, only three institutions completed any form of direct evaluation of clinical counselling skills using role-play or observed interaction with real or standardized patients. Skills training, accompanied by rigorous summative assessments, would be appropriate for training medical students in SCC and ultimately be of benefit to the tobacco users they will undoubtedly encounter in their clerkship and subsequent practice.

Six of the 11 schools surveyed reported devoting between zero and three hours to the topic of pre-clerkship SCC training. Due to the immense burden of tobacco-related diseases on personal and community health and healthcare costs^18^ substantially more time (minimum three to five hours) should be devoted to the topic and include interactive lectures, enhanced methods of learning, practical/clinical skills training and high-quality evaluation.^19^

Given the effectiveness of MI as a direct, patient-centered approach for behavioural modification, academic institutions should aim to incorporate more practical and clinical skills training, employing MI techniques (i.e., role-play, physician skills development, standardized patient interviewing, and practice OSCE cases) in developing and enhancing cessation skills in the pre-clerkship curriculum. Medical schools may incorporate MI training in other areas of the undergraduate curriculum (e.g., addictions management), which could easily be applied to SCC practice. The application of MI skills training, acquired in other domains, to SCC was not formally assessed in our national survey.

There is clear evidence to indicate that all smoking cessation attempts are enhanced by the use of smoking cessation pharmacotherapy.^20^ Medical students should be familiar with evidence-based, best-practice use cessation pharmacotherapies.

Smoking cessation counselling for socio-culturally diverse patients or those with special needs, which may include the disadvantaged, those struggling with mental health issues, members of Indigenous communities, and pregnant smokers, presents its own unique challenges. Effective training in SCC skills should be implemented to ensure medical students are sensitive to the particular needs of special populations.

Only 65% of Canadian medical schools participated in this survey – a limitation of this study. Furthermore, while significant effort was made to identify and locate the most knowledgeable content expert at each institution, it is possible that such experts might not be fully aware of the breadth and depth of tobacco-related curricular content. The possibility, therefore, that curriculum coverage may be over- or under-estimated cannot be excluded. The short survey assessing medical students’ attitudes toward smoking cessation education was only implemented at the University of Ottawa and hence cannot be extrapolated to infer similar attitudes of medical students at other institutions.

In conclusion, this survey of approaches to SCC education for medical students demonstrates substantial deficits and inconsistencies in the delivery of such training in the pre-clerkship curricula of Canadian medical schools. Medical students surveyed at the University of Ottawa expressed a lack of comfort in addressing smoking cessation. These findings suggest potential deficits in tobacco education in Canadian medical schools, comparable to those demonstrated in the U.K in 2015.^9^ Implementing and/or strengthening evidence-based methods of SCC training early in medical school, where they are lacking, is a logical step to strategically address these curriculum deficits. Enhanced inter-university collaboration might facilitate the development of a national, standardized curriculum model to guide undergraduate medical education programs within the pre-clerkship curriculum of Canada’s medical schools. The enormous burden of tobacco disease in our society warrants immediate and concerted effort.
